# Supplementary material for: Social learning about dangerous people by wild jackdaws
Source: R Soc Open Sci. 2019 Sep 25;6(9):191031. doi: 10.1098/rsos.191031 (PMC6774944; doi:10.1098/rsos.191031)
Supplement: Supplementary material for "Social learning about dangerous people by wild jackdaws", Lee et al. (2019) [file rsos191031supp1.pdf]

Social learning about dangerous people by wild jackdaws

Victoria E. Lee[a]*, Noémie Régli[b], Guillam E. McIvor[a] & Alex Thornton[a]*

[a]College of Life and Environmental Sciences, University of Exeter, Penryn Campus, Penryn, Cornwall, UK TR10 9FE.

[b]St Etienne UFR Sciences et Techniques, 23 Rue du Dr Paul Michelon, 42100 Saint-Étienne, France

*Email correspondence: vel202@exeter.ac.uk or A.Thornton@exeter.ac.uk

Supplementary Material

Detailed methods

Playback stimuli

Extracted calls were arranged into playback files comprising either 5 contact calls or 5 scold calls from a single individual, occurring at 2s intervals to simulate natural calling (Figure S1). Where possible, playback files contained 5 different calls from the same individual (contact calls: 3-11 calls from 30 individuals, mean 5 calls per individual; scold calls: 3-13 calls from 18 individuals, mean 4 calls per individual). For cases where fewer than 5 calls were available, the number of repeated calls was kept to a minimum, and order of calls was modified to ensure that test subjects would not hear repeated calls presented in the same order. There were also 2 cases where many high-quality calls were available for an individual, and multiple playback tracks were made (containing different calls from the same individual) to maximise the number of different playback tracks used across the experiment. In total, this produced contact call playback files from 30 individuals (15 males and 15 females, 3-11 calls available per individual) and scold call playback files for 18 individuals (9 males and 9 females, 3-13 calls available per individual, with one male and one female contributing 3 files each).

Behavioural analysis

The close-up view of the nest box from the HD camcorder enabled identification of individuals and recording of behaviours occurring at the nest box. This footage was aligned to the wide-angle footage (using the built-in 'offset' function in BORIS) to allow individuals to be identified as they approached the box. We recorded the frequency and duration of all

behaviours exhibited by either bird during the time the experimenter was sat motionless during the trial (setup times were excluded). Behaviours recorded from the close-up footage included landing on the nest box, entering the nest box, and scolding. From the wide-angle footage, we recorded when birds returned and left the area of the nest box (defined as landing within the view of the wide-angle camera).

An independent coder who was blind to treatment analysed a subset of 15% of the videos. An intra-class correlation coefficient [1] showed a high degree of agreement between coders for the time individuals spent in the nest box (ICC=0.998, p<0.001), latency to enter the nest box (ICC=1,p<0.001), and latency to land on the nest box (ICC=1, p<0.001) (data for females only). The subset of videos for independent coding were selected at random; in some cases, it was initially difficult for inexperienced coders to accurately identify when females returned to the area prior to their first landing (e.g. if birds landed in barns or shaded areas before returning to the nest box). For this reason, the same independent coder analysed another subset of videos for this variable, with all nest boxes located in single trees (10% of the remaining videos, ICC= 0.96, p<0.001). Both coders also confirmed return times for all females in the original subset, once the returning bird had been pointed out by the original coder.

Statistical analysis

Time taken to approach nest box

This model included data from 21 females (10 females in the contact call group; 11 in the scold call group). Of the original sample (n=34), five females were excluded as they were not present during the playback in Phase 2. Seven females did not land on the box during either the baseline (1) or test (3) trials, and due to poor lighting in one trial female return time was

estimated in the video footage. This individual was subsequently excluded from the model after examination of Cook's distances confirmed that this estimate was inaccurate.

Latency to enter nest box

This model included data from 23 females (12 in the contact call group, 11 in the scold call group). In five cases females did not hear the playback during phase 2, and six females did not land on and/or enter the nest box during the baseline (1) or test (3) phase.

Time spent in nest box

20 females were included in this model (12 in the contact call group and 8 in the scold call group), after excluding females that did not hear the playback (n=5) and cases where trials were cut short or disturbed (n=9; although this was not relevant for the latency variables above, disturbance may have influenced the amount of time that females spent in the nest box over the trial as a whole). Females that did not enter the nest box over the course of a trial were given a score of zero.

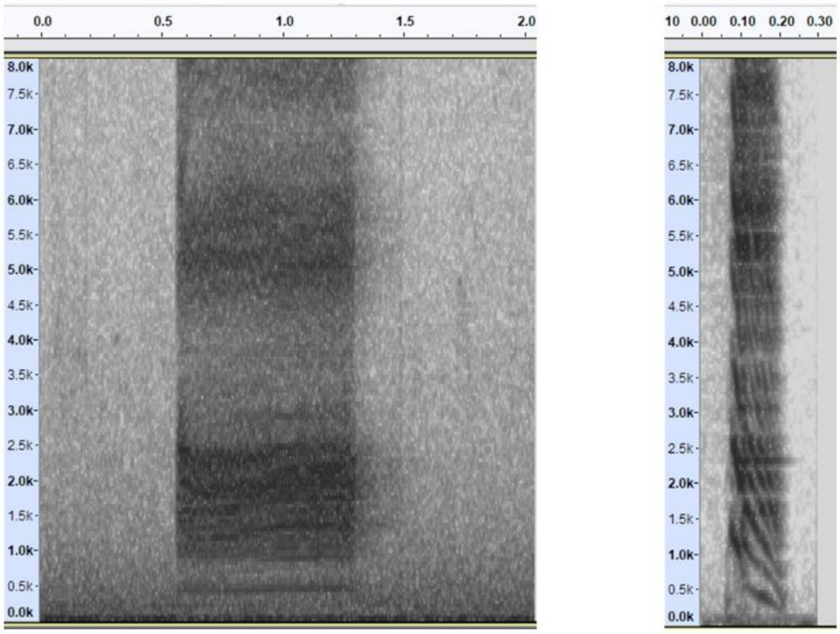

Figure S1: Spectrogram of a jackdaw scold call (left) and a jackdaw contact call (right). Playbacks were comprised of 5 calls from the same individual separated by an inter-call interval of 2 seconds to simulate natural call rates.

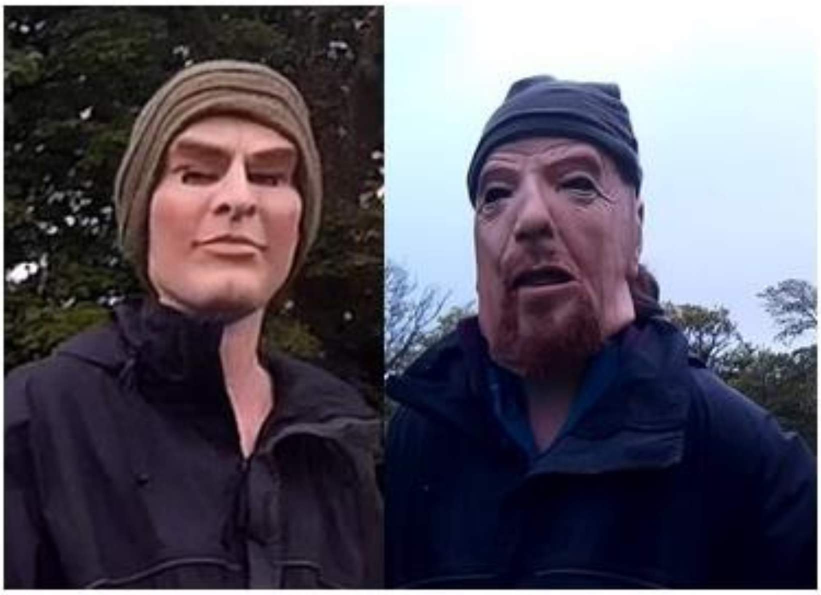

Figure S2: Masks used by experimenters during trials. Each focal jackdaw pair was given three presentations of the same experimenter wearing the same mask. Masks were paired with a plain hat and the same clothing was worn by experimenters throughout the trials. Each mask was allocated to a consistent playback treatment (scold calls/contact calls) at a given field site, and mask-treatment allocations were counterbalanced between sites.

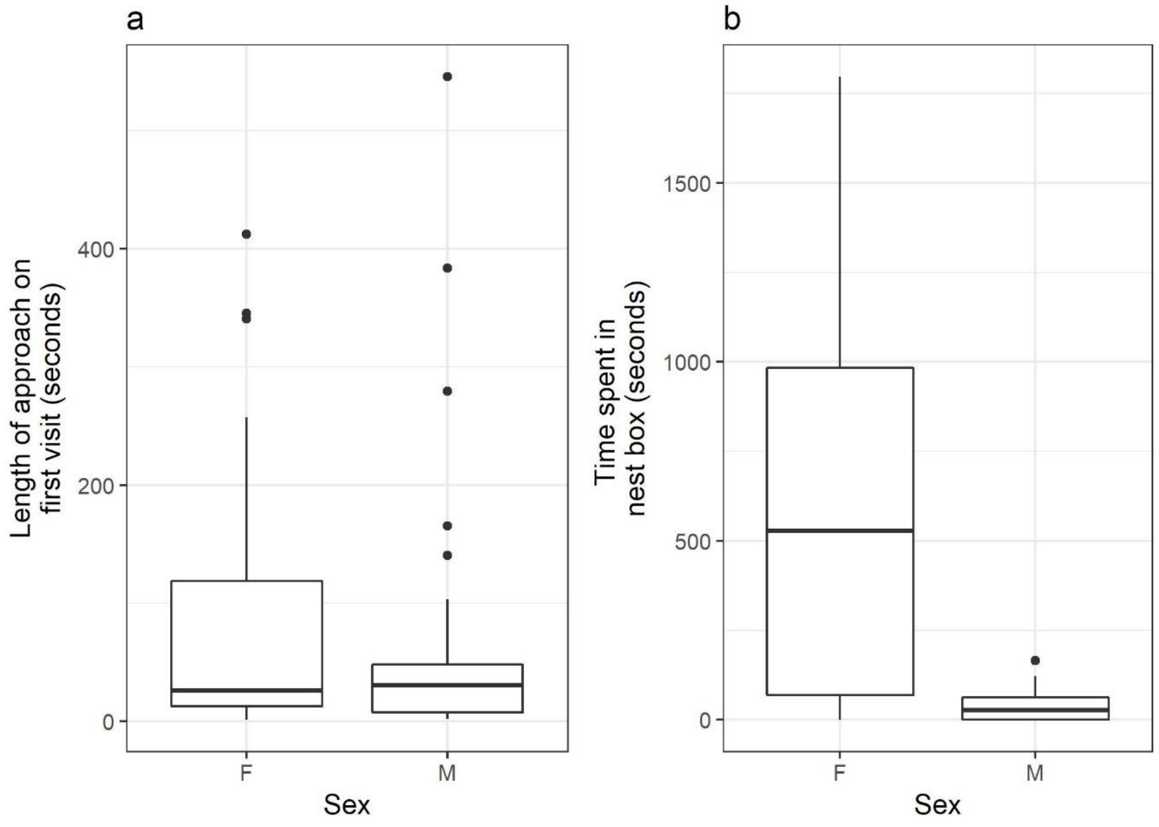

Figure S3: Heterogeneity of variance between sexes for key behavioural responses. a) Females were more variable than males in their latency to approach the nest box on the first visit; b) Females spent more time in the nest over the course of the trials, as expected at this stage in the breeding attempt.

Table S4: Summary statistics for female approach times on their first visit to the box in the baseline and test phases, by treatment group.

	**Scold Calls**		**Contact Calls**	
	Baseline	Test	Baseline	Test
n	11	11	10	10
Mean±SE	92.7s ± 31.0	43.6s ± 23.2	70.6s ± 26.5	114.8s ± 37.2
Median±IQR	65.6s ± 104.2	13.3s ± 8.8	31.1s ± 26.2	62.6s ± 177.2

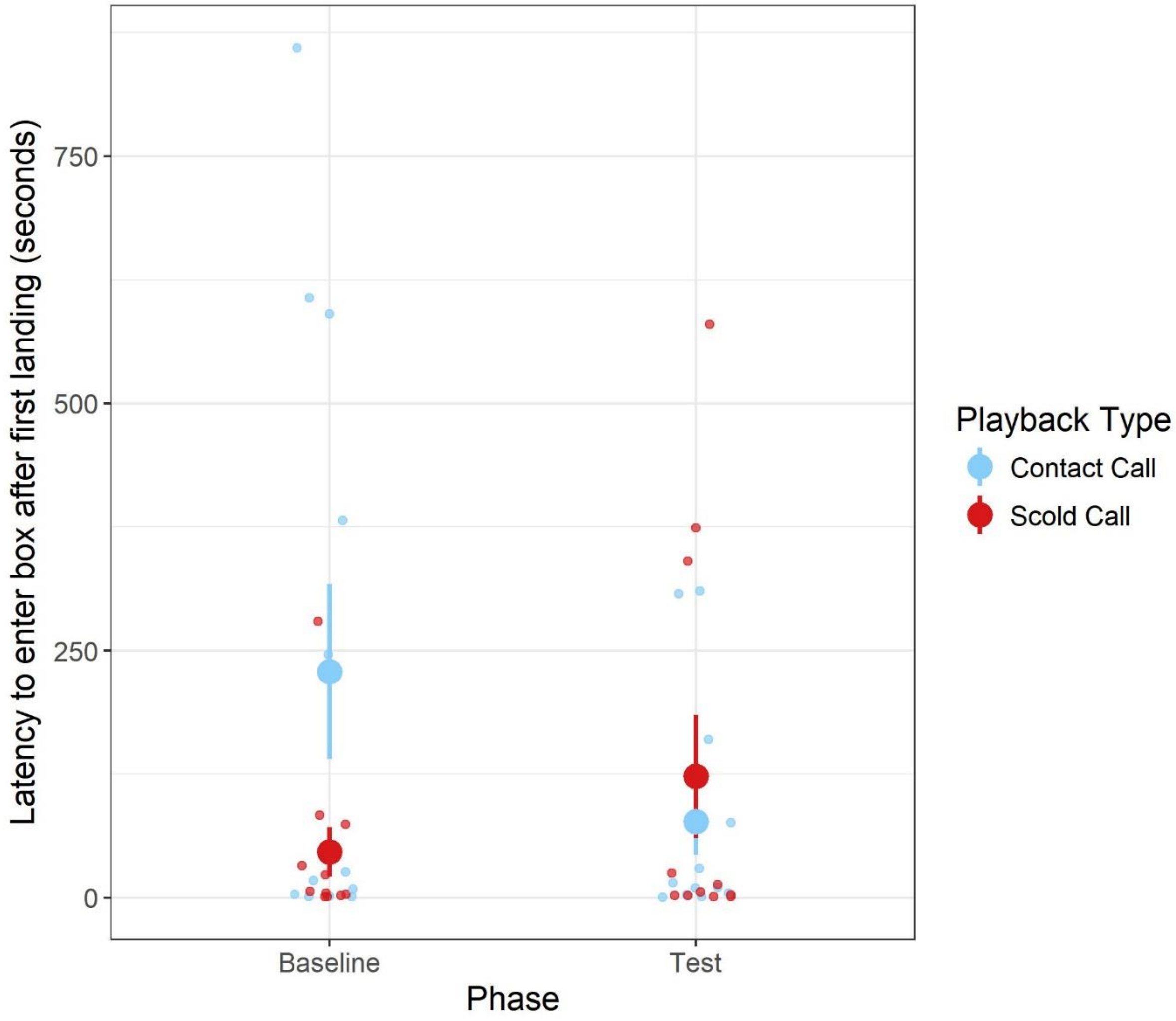

Figure S5: Female latency to enter after first landing on the nest box in the baseline (trial 1) and test phase (trial 3), according to playback type (scold calls/contact calls). Points and whiskers denote mean and standard error, n=46 observations from 23 females.

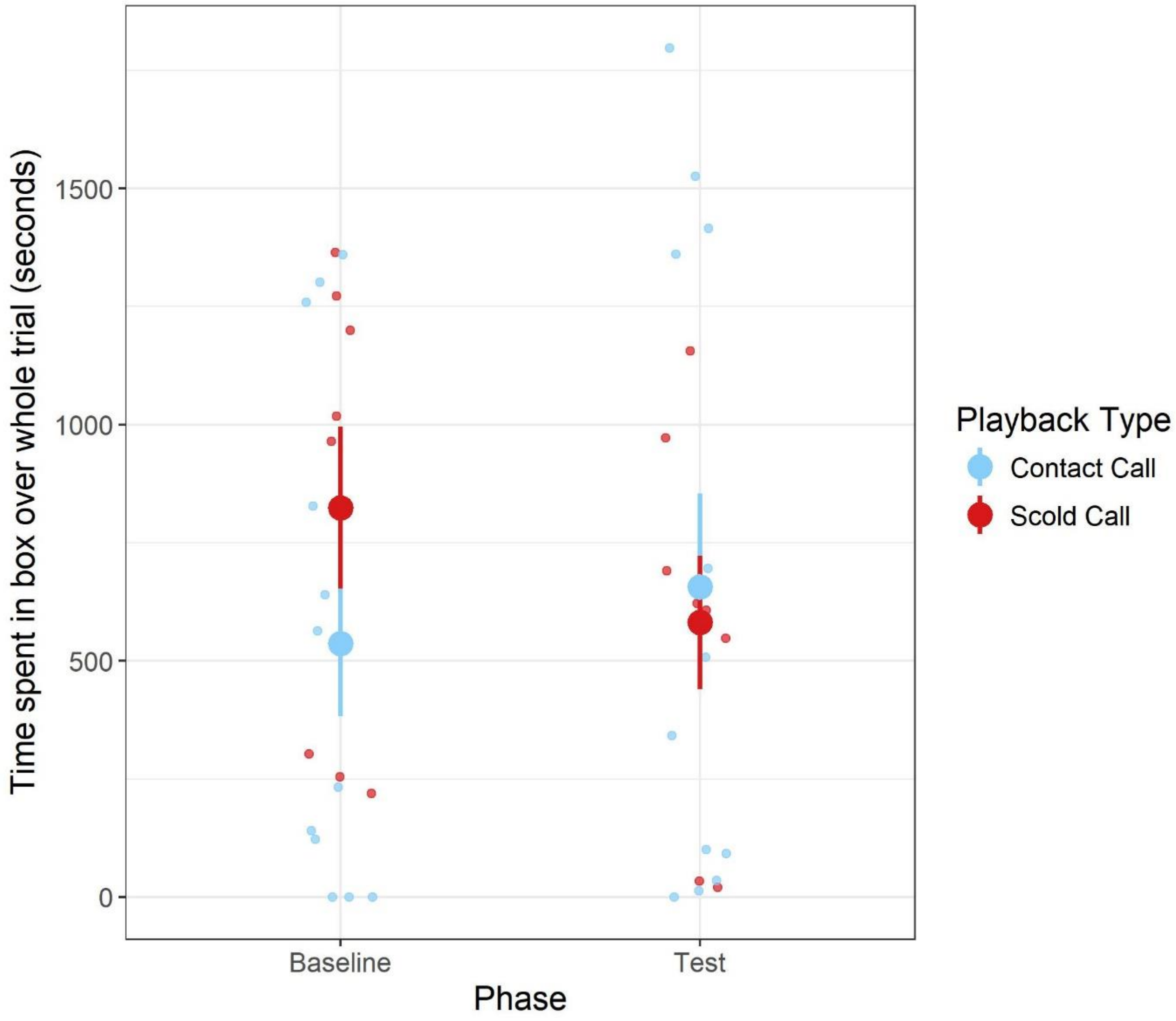

Figure S6: Time spent by females in the nest box during baseline (trial 1) and test phase (trial 3), according to playback type (scold calls/contact calls). Points and whiskers denote mean and standard error, n=40 observations from 20 females. Females that did not enter the nest box during the trial were given a score of zero.

Table S7: Proportion of females scolding in each experimental treatment (infrequent scolding precluded formal analysis of this response). Excludes individuals that were not present during the playback presentation in the training phase.

Proportion of females scolding	**Baseline**	**Test**
**Contact calls**	4/16	2/16
**Scold calls**	1/13	1/13

References

1. Gamer M, Lemon J, Fellows I, Singh P. 2012 irr: various coefficients of interrater reliability and agreement. R package version 0.84.
